# Heliox Allows for Lower Minute Volume Ventilation in an Animal Model of Ventilator-Induced Lung Injury

**DOI:** 10.1371/journal.pone.0078159

**Published:** 2013-10-18

**Authors:** Charlotte J. Beurskens, Hamid Aslami, Friso M. de Beer, Margreeth B. Vroom, Benedikt Preckel, Janneke Horn, Nicole P. Juffermans

**Affiliations:** 1 Laboratory of Experimental Intensive Care and Anaesthesiology, Academic Medical Center University of Amsterdam, Amsterdam, The Netherlands; 2 Department of Anaesthesiology, Academic Medical Center, University of Amsterdam, Amsterdam, The Netherlands; 3 Department of Intensive Care, Academic Medical Center, University of Amsterdam, Amsterdam, The Netherlands; University of Giessen Lung Center, Germany

## Abstract

**Background:**

Helium is a noble gas with a low density, allowing for lower driving pressures and increased carbon dioxide (CO_2_) diffusion. Since application of protective ventilation can be limited by the development of hypoxemia or acidosis, we hypothesized that therefore heliox facilitates ventilation in an animal model of ventilator–induced lung injury.

**Methods:**

Sprague-Dawley rats (N=8 per group) were mechanically ventilated with heliox (50% oxygen; 50% helium). Controls received a standard gas mixture (50% oxygen; 50% air). VILI was induced by application of tidal volumes of 15 mL kg^-1^; lung protective ventilated animals were ventilated with 6 mL kg^-1^. Respiratory parameters were monitored with a pneumotach system. Respiratory rate was adjusted to maintain arterial pCO_2_ within 4.5-5.5 kPa, according to hourly drawn arterial blood gases. After 4 hours, bronchoalveolar lavage fluid (BALF) was obtained. Data are mean (SD).

**Results:**

VILI resulted in an increase in BALF protein compared to low tidal ventilation (629 (324) vs. 290 (181) μg mL^-1^; p<0.05) and IL-6 levels (640 (8.7) vs. 206 (8.7) pg mL^-1^; p<0.05), whereas cell counts did not differ between groups after this short course of mechanical ventilation. Ventilation with heliox resulted in a decrease in mean respiratory minute volume ventilation compared to control (123±0.6 vs. 146±8.9 mL min^-1^, P<0.001), due to a decrease in respiratory rate (22 (0.4) vs. 25 (2.1) breaths per minute; p<0.05), while pCO_2_ levels and tidal volumes remained unchanged, according to protocol. There was no effect of heliox on inspiratory pressure, while compliance was reduced. In this mild lung injury model, heliox did not exert anti-inflammatory effects.

**Conclusions:**

Heliox allowed for a reduction in respiratory rate and respiratory minute volume during VILI, while maintaining normal acid-base balance. Use of heliox may be a useful approach when protective tidal volume ventilation is limited by the development of severe acidosis.

## Introduction

In acute respiratory distress syndrome (ARDS), aerated lung volume is diminished [1]. Regression of healthy lung parts and increased airway resistance results in overdistention of non-injured alveoli, even during lung protective ventilation with limited tidal volume and plateau pressures [2], thereby contributing to ventilator-induced lung injury (VILI). The only proven strategy aimed at limiting lung injury is low tidal volume ventilation [3]. However in patients with severe ARDS, tidal volumes and pressures that exceed recommendations of the ARDS network are often applied [4]. Adjunctive therapies such as extracorporeal CO_2_ removal have been shown to allow for a further decrease in tidal volume ventilation with additional protection in ARDS [5,6]. However, use of these devices come with a certain complication rate and require expertise. Thereby, other therapeutic strategies are needed that allow for a decrease in peak pressures and minute volume ventilation for adequate gas exchange, which may facilitate adherence to protective ventilation strategies. 

Helium is an inert gas with a lower density than air [7], allowing for less turbulent flow through airways, leading to lower airway resistance. As a result, during mechanical ventilation with a helium/oxygen mixture (heliox), lower driving pressures are needed to distribute oxygen to the distal alveoli compared to ventilation with oxygen [8]. Also, diffusion of CO_2_ is increased during heliox, which in addition might facilitate ventilation.

The use of heliox is clinically applied in patients with high airway resistance due to exacerbations of chronic obstructive pulmonary disease (COPD) or status asthmaticus [9,10,11]. In paediatric animal models of ARDS [12,13,14], heliox ventilation was found to improve gas exchange during high–frequency oscillatory ventilation and attenuate lung inflammation [13]. However, data on the effect of heliox on ARDS during conventional mechanical ventilation are sparse and data are limited to paediatric models. 

In this study we investigated the effects of heliox in an adult rat VILI model. We hypothesized that the use of heliox facilitates CO_2_ elimination allowing for lower minute volume ventilation. Furthermore, the effect of heliox on the inflammatory response was investigated. 

## Materials and Methods

The animal care and use committee of the Academic Medical Centre, University of Amsterdam, Netherlands approved this study. Animal procedures were carried out in compliance with Institutional Standards for Use of Animal Laboratory Animals. Male Sprague–Dawley rats (Harlan, The Hague, The Netherlands), weighing 350–400 grams were anaesthetized by intraperitoneal injection of 90 mg kg^-1^ ketamine, 0.125 mg kg^-1^ dexmedetomidine and 0.05 mg kg^-1^ atropine. Anaesthesia was maintained by infusion of 10 mg mL^-1^ ketamine at 2.7 mL per hour. A solution of saline and bicarbonate was administered at 2.5 ml per hour. A tracheotomy was performed and a metal cannula was inserted in the trachea. Two sutures were placed around the exposed part of the trachea into which the cannula was inserted and tied down thoroughly. The cannula was then connected to a ventilator (Servo 900C, Siemens, Sweden). The ventilators were calibrated for the heliox gas mixture according to the instruction of the manufacturer using a pressure reduction valve to allow the high-pressure of the heliox tank to be reduced to safe and usable pressures for ventilation (Linde Gas Therapeutics, Eindhoven, the Netherlands). Hemodynamic parameters were monitored by inserting a polyethylene catheter into the carotid artery connected to a monitor (Siemens SC900, Danvers, USA). Temperature was monitored rectally and maintained at 37°C by a thermo mattress.

VILI was induced by application of tidal volumes of 15 mL kg^-1^ and zero PEEP during 4 hours. Lung protective (LP) ventilation was maintained by applying 6 mL kg^-1^ and 5 cmH_2_O Peep. FiO_2_ was set at 50%, I:E ratio of 1:2 and adjustment of respiratory rate to maintain PaCO_2_ within 4.5–5.5 kPa, according to hourly drawn arterial blood gases. The alveolar-arterial gradient was calculated as follows: A − a gradient = (Fraction of inspired oxygen (%)/100) × (P_Atmosphere_ − P_H2O_) − (PaCO_2_/0.8) − PaO_2_. Dead space was calculated using the formula: Dead space = (PaCO_2_-etCO_2_)/ (PaCO_2_)*100.

Rats were ventilated pressured controlled, with either heliox (technical gas 50% oxygen; 50% helium; blended by Linde Gas Therapeutics) or 50% oxygen in air gas mixture (n=8 per group). In total 32 animals were studied of which 16 received heliox (8 VILI; 8 LP ventilation) and 16 received 50 % oxygen in air gas mixture (8 VILI; 8 LP ventilation).

Tidal volumes were strictly maintained using a pneumotachometer (Hugo Sachs Elektronik, Harvard apparatus, March–Hugstetten, Germany) specific for rats. The pneumotachometer is a transducer for airflow measurement, placed between the metal cannula and the ventilator, with a minimum dead space (50 µL). The pneumotachometer was calibrated using a 1 mL syringe according to the manufacturer’s instruction. Tidal volumes were recorded using respiration software (HSE-BDAS basic data acquisition, Harvard apparatus, March–Hugstetten, Germany) and displayed on a computer screen throughout the whole experiment. Since small tidal volumes are delivered it is not possible to ventilate the animals in a volume-controlled setting. We set a pressure controlled ventilation mode and started with an inspiratory pressure of 10 cmH_2_O. In case of a deviation of the set tidal volume, the inspiratory pressure was adjusted [15,16,17]. Compliance was calculated by dividing tidal volume per kilogram by inspiratory pressure. 

After 4 hours of mechanical ventilation, rats were bled and plasma was centrifuged at 1800g for 10 minutes at 4°C. Lungs were removed en block. After the right lung was ligated, bronchoalveolar lavage was done by flushing the left lung 3 times with 2.0 ml NaCl, yielding about 5 ml of lavage fluid. In the bronchoalveolar lavage fluid (BALF), cells were counted using a hematocytometer (Z2 Coulter Particle Counter, Beckman Coulter Corporation, Florida). After centrifugation of BALF (300g; 10 min.; 4°C), protein levels were measured (Oz Biosciences, Marseille, France) and levels of Interleukin (IL)-1β, IL–6, cytokine–induced neutrophil chemo attractant (CINC)-3, and Tumor Necrosis Factor (TNF)-α were determined by ELISA, according to instructions of the manufacturer (R&D Systems; Abingdon, United Kingdom). 

### Statistical analysis

Data are expressed by mean and SEM. The effect of heliox versus oxygen within either the VILI or the LP group was compared using a one-way ANOVA or Kruskal Wallis test, with either a Bonferroni's or Dunn's multiple comparison test, depending on distribution of the data. Statistical significance was set at *P*<0.05. 

## Results

All animals survived the experimental protocol. Mean arterial pressure remained above 90 mmHg in all groups. Heliox did not affect blood pressure or heart rate compared to the oxygen-ventilated animals. 

### VILI model

**Figure 1:**
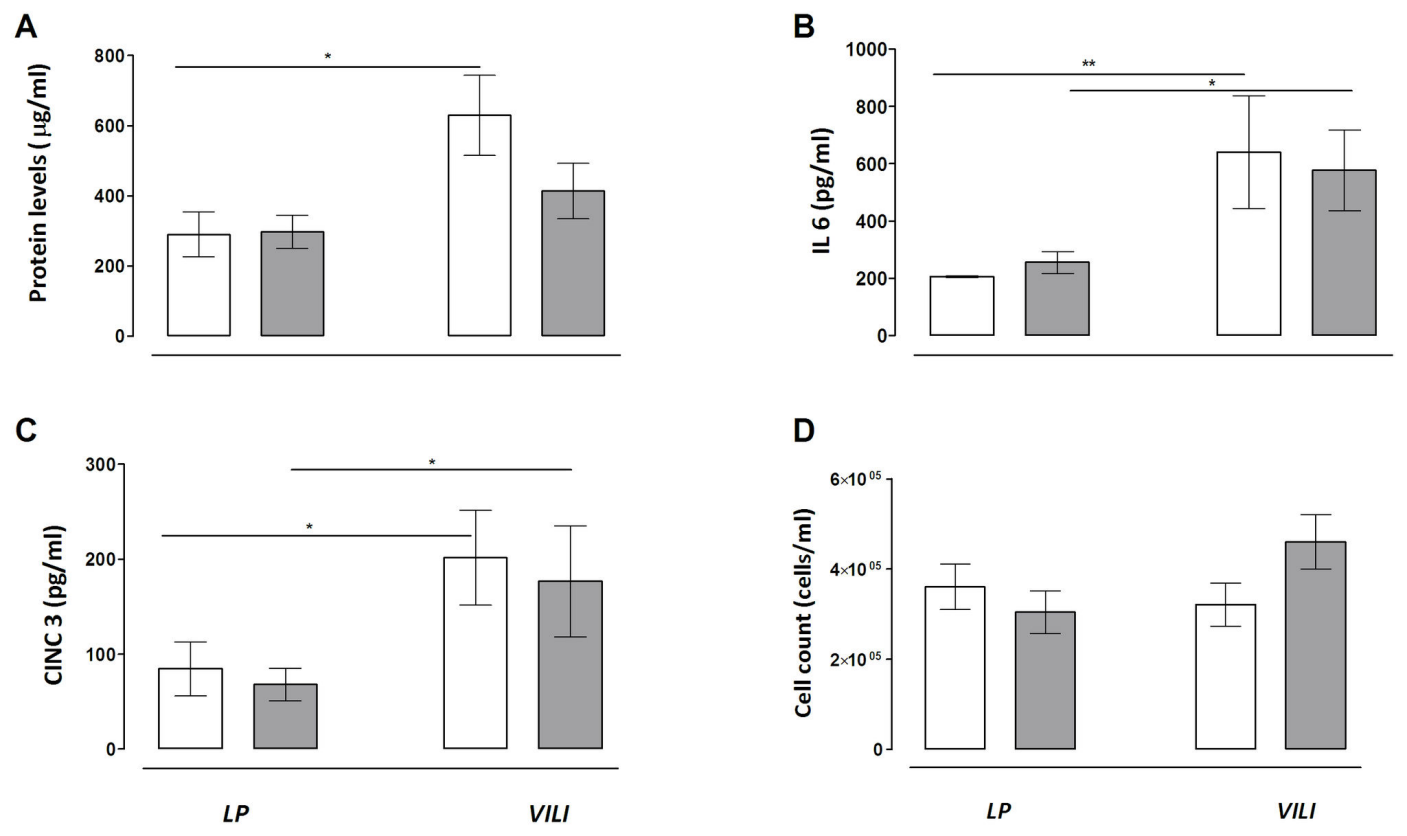
Inflammatory parameters in an animal model of ventilator–induced lung injury (N=8 per group), treated with heliox ventilation. Heliox is marked by white bars and oxygen/air by grey bars. Data are MEAN ± SEM. *: P < 0.05; **: P < 0.01. (A) Protein levels; (B) IL- 6 levels; (C) CINC–3 levels and (D) cell count in bronchoalveolar lavage fluid.

**Figure 2:**
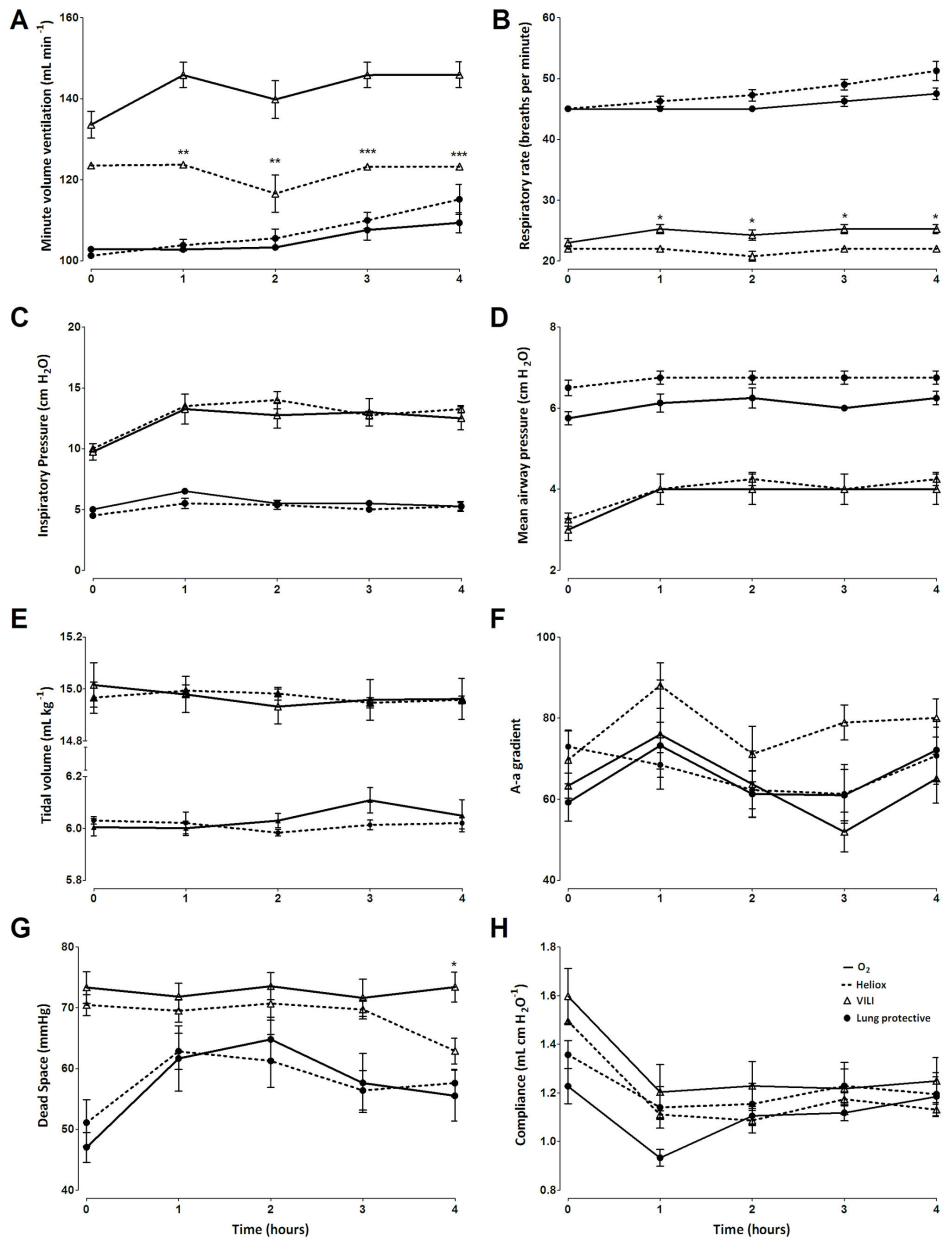
Respiratory parameters in an animal model of lung injury during treatment with heliox ventilation (N=8 per group). Data are MEAN ± SEM. VILI is marked by open triangles; LP ventilation is marked by filled circles. Heliox ventilation is marked by a disconnected line and oxygen/air by a continuous line. Comparisons are between heliox and oxygen within the VILI or the LP group. *: P < 0.05; **: P < 0.01; ***: P < 0.001. (A) Minute volume ventilation (mL min^-1^); (B) respiratory rate (breaths per min); (C) inspiratory pressure (cm H_2_O); (D ) mean airway pressure (cm H_2_O); (E ) tidal volume (mL kg^-1^); (F) A-a gradient; (G) dead space (mmHg) and (H) compliance (mL cm H_2_O ^-1^).

Application of high tidal volumes resulted in VILI, characterized by an increase in mean pulmonary protein leakage and BALF levels of IL–6 and CINC–3 (Figure 1). Pulmonary cell influx was not increased in VILI compared to controls, nor was the BALF level of IL1–β and TNF–α (data not shown). Applied respiratory rate needed to keep PaCO_2_ within predefined limits (4.5-6 kPa) increased during mechanical ventilation in both VILI groups, yielding higher minute volume ventilation over time, while compliance decreased over time (Figure 2). 

### Effects of heliox

Ventilation with heliox resulted in a decrease in minute volume ventilation compared to oxygen ventilation, due to an adjustment in respiratory rate, while tidal volumes were kept at 6 mL kg^-1^ according to protocol (Figure 2). PaCO_2_ levels and pH remained unchanged (table 1); according to the study protocol in which set arterial PaCO_2_ levels were targeted. Heliox did not affect mean airway pressure, peak and inspiratory pressure or compliance (Figure 2). A-a gradients showed no differences (Figure 2). Dead space in VILI was reduced by heliox after 4 hours (Figure 2). Heliox ventilation did not decrease any of the parameters of lung inflammation, including pulmonary oedema, BALF protein or cytokine levels (Figure 1). 

**Table 1:** Gas exchange and respiratory parameters at baseline and after 4 hours of injurious mechanical ventilation in an animal model of lung injury during and after treatment with heliox ventilation.

		**LP**		**VILI**	
	**Time (hr)**	**Oxygen**	**Heliox**	**Oxygen**	**Heliox**
pH	T = 0	7.44 ± 0.04	7.41 ± 0.03	7.42 ± 0.08	7.43 ± 0.04
	T = 4	7.33 ± 0.04	7.36 ± 0.03	7.38 ± 0.10	7.40 ± 0.05
pCO_2_ (kPa)	T = 0	4.38 ± 0.7	4.86 ± 0.6	5.09 ± 1.0	4.98 ± 0.3
	T = 4	4.99 ± 0.4	5.81 ± 0.9	5.07 ± 1.1	4.76 ± 0.5
pO_2_ (kPa)	T = 0	34.2 ± 1.8	31.7 ± 1.5	32.7 ± 1.9	32.0 ± 2.6
	T = 4	31.7 ± 2.5	30.8 ± 2.8	32.5 ± 3.1	30.9 ± 2.2
etCO_2_ (mmHg)	T = 0	16.7 ± 2.1	17.6 ± 2.9	10.0 ± 0.6	11.2 ± 1.6
	T = 4	16.7 ± 3.6	18.2 ± 2.2	10.0 ± 1.3	12.3 ± 1.6

(N=8 per group).

Data are MEAN ± SD.

LP = lung protective VILI = ventilator induced lung injury

## Discussion

In a rat VILI model, increased minute volume ventilation was needed to maintain adequate gas exchange. Heliox allowed for a reduction in minute volume ventilation, while CO_2_ removal remained unaltered. These effects are in line with findings in pediatric ARDS models [12,13,14], as well as in pediatric and adult patients with respiratory failure due to exacerbation of COPD or asthma [9,10,18,19,20]. In ARDS models, heliox has mostly been studied as a driving gas during HFOV. In this setting, heliox improved CO_2_ elimination, which was found to be due to an increase in tidal volume delivery, thereby hampering the use in clinical practice in ARDS patients [14,21]. In our study, ventilation improved at unchanged tidal volume delivery during conventional pressure controlled mechanical ventilation settings. 

The theory that heliox ventilation may allow for lower peak inspiratory pressures in ARDS patients has been postulated before [11], as heliox establishes a more laminar flow through small airways compared to oxygen [7,8]. In our study however, heliox did not allow for a reduction in inspiratory pressures applied to achieve pre set tidal volumes. Thereby, compliance of the respiratory system also remained unchanged. This is in line with a study in children with acute bronchiolitis due to infection with respiratory syncytial virus, in which heliox decreased airway resistance at unchanged tidal volume delivery during pressure controlled ventilation, but did not allow for application of lower peak pressures [22]. Whether helium has therapeutic potential in ARDS, by increasing ventilation in order to increase adherence to protective ventilation strategies including low tidal volume ventilation and use of limited inspiratory pressures, remains to be determined. 

Ventilation with heliox did not attenuate pulmonary inflammation in VILI. These results are in contrast with previous findings in models of ARDS in which an anti-inflammatory effect of heliox has been described [13,23]. There may be several explanations for these disparate results. In neonatal pigs, heliox was found to reduce tidal volumes with a concomitant decrease in inflammation [13]. In that study, it was not clear whether heliox exerted direct anti-inflammatory effects or that inflammation was reduced due to less mechanical stress [13,23,24]. In a rat model in which ARDS was induced by saline lavage during pressure controlled ventilation, use of 80% heliox decreased inflammation, already after 1 hour of ventilation [23]. In this study, tidal volumes were not measured. Possibly, the higher concentration of heliox may have been beneficial. In our study, we choose to keep tidal volumes constant during the experiment while adjusting respiratory rate to maintain an adequate gas exchange, in order to dissect whether reduction of lung injury was due to heliox ventilation alone and not due to application of tidal volumes lower than 6 mL kg^-1^. As tidal volume is a clear risk factor for ARDS [25,26] it is possible and even probable that further reducing tidal volumes during heliox ventilation would result in reduced lung injury. 

An alternative explanation for the absence of an anti-inflammatory effect may be that our VILI model is mild. Although pulmonary protein leak and cytokine levels differed between VILI and LP controls, pulmonary edema and cell influx were unaltered. Of note, these tidal volumes are used in daily practice, so therefore the model is relevant to investigate the effect of gas mixtures on inflammatory and respiratory parameters [26]. Taken together, we conclude that in this relevant VILI model, heliox does not have anti-inflammatory properties. In line with this, heliox did not alter immune response of healthy volunteers stimulated ex vivo with various bacterial stimuli [27].

## Conclusions

Heliox ventilation allowed for decreased minute volume ventilation in this rat VILI model. Whether helium has therapeutic potential in ARDS, by increasing adherence to protective ventilation strategies including lowering of tidal volume and use of limited inspiratory pressures, requires further investigation. 
